# Influence of arousal on intentional binding: Impaired action binding, intact outcome binding

**DOI:** 10.3758/s13414-020-02105-z

**Published:** 2020-09-30

**Authors:** Anna Render, Petra Jansen

**Affiliations:** Faculty of Human Science, University of Regensburg, Universitätstraße 31, 93053 Regensburg, Bavaria Germany

**Keywords:** Sense of agency, Intentional binding, Action binding, General arousal, Sexual arousal

## Abstract

Emotional states have been indicated to affect intentional binding, resulting in an increase or decrease as a function of valence and arousal. Sexual arousal is a complex emotional state proven to impair attentional and perceptual processes, and is therefore highly relevant to feeling in control over one’s actions. We suggest that sexual arousal affects intentional binding in the same way as highly negative arousing states such as fear and anger. Ninety participants performed the intentional binding task before and after watching an either sexually arousing or emotionally neutral film clip. Analyses were conducted for the subcomponents action and outcome binding separately including the change in arousal before and after the emotion induction as a continuous measure. Results showed an interactive effect for time of measurement (before and after emotion induction) and arousal change on action binding: a decrease in action binding was noted in participants who reported to be more aroused and an increase in action binding was observed for participants who reported to be less aroused. Results emphasize that alterations in action binding are likely to reflect the deficits in the dopaminergic system involved in action execution. An impaired feeling of control in aroused states may play a crucial role for the underlying psychological mechanisms of impulsive violent behavior.

**Electronic supplementary material:**

The online version of this article (10.3758/s13414-020-02105-z) contains supplementary material, which is available to authorized users.

## Introduction

### Intentional binding

The awareness of control over one’s own actions and naming the cause of action is referred to as the *sense of agency* (Gallagher, 2000). The sense of agency is essential to explain changes in the external world and for a foundation for one’s future predictions (Wen, Yamashita, & Asama, 2015). The degree of consciousness of actions can be measured with an implicit paradigm called intentional binding. Intentional binding defines a time shift in the perception between a voluntary executed action and a following sensory event. If an action feels controlled, a binding effect between action and event can be observed: the interval is perceived as shorter than it really is; in other words, a subjective compression of time occurs (Haggard, Clark, & Kalogeras, 2002). This is indicated by a forward shift of an action toward its outcome (action binding) and a backward shift of an outcome towards its action (outcome binding) (Lush et al., 2019).

Intentional binding can be measured with two different tasks, the Libet clock and the interval estimation (also interval reproduction) task. While time estimations for actions and outcomes in the Libet clock task are made in reference to the hand of an analogue clock (rotating faster than a usual clock), the interval-estimation task works without such a visual reference. Thus, time estimations are a reproduction of the subjective length of a previously experienced event (e.g., Dewey & Knoblich, 2014).

#### Subcomponents of intentional binding

Providing cues for our judgments of actions and consequences, the Libet clock task offers insight into the differences of action and outcome binding, since these are at least partly driven by distinct mechanisms. A recent meta-analysis, taking 78 studies into account, found evidence for this assumption: Action binding is more dependent on whether one can control outcome onsets with voluntary actions; outcome binding depends more on the degree to which participants can predict, rather than control, the outcome onset (Tanaka, Matsumoto, Hayashi, Takagi, & Kawabata, 2019). Hence, interpreting action and outcome binding separately provides more information about the underlying mechanisms and processes regulating them (Wolpe & Rowe, 2014), which could contribute to the understanding of abnormal experience of agency (Tanaka et al., 2019).

Considering that both subcomponents are driven by distinct mechanisms, it stands to reason to examine effects of experimental manipulations such as inducing an emotional state on action and outcome binding separately.

### Manipulation of emotional states during action performance

The state of control of an individual has been shown to be determined by their emotional state during the performance of actions (Christensen, Di Costa, Beck, & Haggard, 2019). To this point, studies manipulating the emotional state whilst performing an action have either reported results for intentional binding as an overall score or for action binding only.

#### Influence on (overall) intentional binding

With regard to the influence of being emotionally neutral aroused on intentional binding, it was reported that highly general aroused participants showed an enhanced implicit binding process between actions and outcomes (a stronger *overall* intentional binding) measured with the interval-estimation task (Wen et al., 2015) compared to participants who were not aroused. Importantly, this enhancement was not evoked by changes in time perception. However, a higher state of arousal had no influence on subjective agency ratings. These findings emphasize the notion that subjective judgment through self-reports and the intentional binding effect seem to reflect different facets of the construct sense of agency (Dewey & Knoblich, 2014; Moore, Middleton, Haggard, & Fletcher, 2012). Wen et al. (2015) therefore suggested that the intentional binding effect involves predictive and inferential processes, inferring that arousal only enhances the predictive process. It must be noted that the arousal manipulation was tested in a second experiment with ten different participants, whereas no information about arousal check was provided for the participants in the actual intentional binding experiment limiting the interpretation of results.

#### Influence on action binding

These results were expanded for emotionally negative arousing states (Christensen et al., 2019) – fear and anger – driven by the aim to simulate situations in court dealing with loss of control in legal defense. It was argued that fear and anger are assumed to attenuate the responsibility over one’s own actions, although the effects of negative aroused states on sense of agency had not been investigated until then. The defendant’s emotional state prior to and during their action performance is more likely to be considered for the sentence than the emotional quality of the outcome; therefore, it was tested if negative emotional states would reduce action binding. Action binding is specific to conditions in which an action is internally generated and executed voluntarily (Borhani, Beck, & Haggard, 2017). In this sense, action binding provides a direct measure showing how close the mental representation of an action is linked to the action’s outcome (Christensen et al., 2019). Thus, in this experiment, fear was induced by moderately painful shocks and anger was generated by a frustration task, in which successful performance on the assignment was impossible. The negative emotion reflected the participant’s emotional state at the time of acting and was not linked to any specific events in the action binding trials. In both emotional states, the impact of fear and anger reduced action binding. A possible inference for this reduction is a psychological distancing from outcomes since they are linked to a negative valence.

### Underlying processes of violent sexual behavior

Psychological distancing from action outcomes and a reduced feeling of control over actions may play a crucial role in violent sexual behavior. It has been pointed out that risk-need treatment approaches tend to ignore the role of personal agency by focusing only on causes such as dynamic risk factors, although we feel the need to seek meaning in and reasons for our actions (Ward & Gannon, 2006). Judgments relating to actions have received little attention in research, although they are important concepts in an individual's insight about their sexual offending. This is why some researchers strengthen the importance of seeing people who have sexually offended as agents actively forming their lives (Ward, Gannon, & Keown, 2006). In fact, it has been reported from the offender’s perspective that people assigned significantly more attention to a feeling of agency compared to other factors such as interpersonal relatedness at the time of their sexual offending (Barnett & Wood, 2008), underlining the importance of sense of agency in sexual arousal.

### Composition of sexual arousal

Research focusing on the constitution of sexual arousal in a laboratory setting has indicated that it not only overlaps with many positive emotions, but also with negative emotions (Everaerd & Kirst, 1989) such as anxiety and anger (Barclay, 1969; Barlow, Sakheim, & Beck, 1983; Beck, Barlow, Sakheim, & Abrahamson, 1987; Wolchik et al., 1980). This has been confirmed in physiological responses (Janssen, Everaerd, van Lunsen, & Oerlemans, 1994; Laan, Everaerd, van Bellen, & Hanewald, 1994), highlighting that sexual stimuli elicit complex responses (Janssen, Everaerd, Spiering, & Janssen, 2000). This overlap of positive and negative emotions in sexual arousal categorizes it as an ambivalent emotional state (Peterson & Janssen, 2007), which has implications for the influence on perception and cognition.

### Influence of sexual arousal on perception and cognition

There is a body of research examining the negative effects of sexual arousal on other cognitive processes such as perception (Most, Smith, Cooter, Levy, & Zald, 2007) or memory (Mather & Sutherland, 2011). It has also been shown that experiencing lower inhibitions in sexual arousal affects predictions of the individual’s own judgments, decision-making processes and behavior, self-control, and sexual self-restraint (Ariely & Loewenstein, 2006; Ditto, Pizarro, Epstein, Jacobson, & MacDonald, 2006; Skakoon-Sparling, Cramer, & Shuper, 2016; Skakoon-Sparling & Cramer, 2016) – processes that are likely to share mechanisms with intentional binding.

Pleasant erotic distractors have been proclaimed to elicit a temporary “emotion-induced blindness” in perceptual processes. Thus, erotic stimuli have been revealed to be distracting and cannot be ignored, confirming that a deficit in perceptive processing can be evoked by positively arousing stimuli to the same extent as by aversive stimuli (Most et al., 2007). Moreover, regardless of the valence, arousing stimuli, such as erotica and mutilation, affect attentional selectivity measured in binocular rivalry (Sheth & Pham, 2008), in attentional blink (Keil & Ihssen, 2004), and in event-related potentials (Schupp et al., 2007).

### Goals and hypotheses

Previous research has provided evidence for an increased intentional binding in generally aroused states (Wen et al., 2015). However, it has to be considered that general arousal differs from sexual arousal: general arousal has been claimed to enhance cognitive processes to a certain degree (e.g., Wen et al., 2015), whereas sexual arousal, as a high arousing but ambivalent state, is assumed to act as an inhibitor of cognitive processes (e.g., Most et al., 2007). Research in this field suggests that it captures attention impairing other cognitive processes in a similar manner as arousing states with negative valence (Most et al., 2007). We therefore expect sexual arousal to impair binding.

Furthermore, as in previous studies (Christensen, Yoshie, Di Costa, & Haggard, 2016; Christensen et al., 2019), we expect to see alterations in action binding only and not in outcome binding. This stands to reason as action binding is known to be specific to conditions where an action is internally generated and executed voluntarily (Borhani et al., 2017), providing a direct measure to show how close the mental representation of an action is linked to its outcome (Christensen et al., 2019). On the basis of the meta-analysis of Tanaka et al. (2019), it can be concluded that action and outcome binding are, respectively, driven by both predictive and inferential processes, but they show different patterns in their underlying mechanism, which is why the two subcomponents should be examined separately. Action binding captures a specific impairment in action planning or generating an action outcome prediction (Tanaka et al., 2019). An intense emotional state might impair the preciseness of these prediction processes. From this, we expect sexual arousal to decrease action binding whilst not affecting outcome binding.

## Materials and methods

### Participants

Ethical approval for the study was obtained from the Research Ethics Committee of the University of Regensburg (project code 18-1203-101), prior to commencement of any testing activities. Participants were informed about the purpose of the study and gave their written consent prior to participation.

In total 90 individuals participated in this study and were pseudo-randomly distributed considering gender balance to one of three groups. Analyses were conducted on de-identified data. Eighty-nine participants provided information on their age, which ranged from 18 to 29 years, *M* = 21.72, *SD* = 2.11; one participant chose not to answer. Thirty-nine (43.3%) participants identified as male, 51 (56.7%) as female; 89 participants classified themselves as heterosexually oriented, and one female participant reported being bi-sexually oriented.

The design included three groups, a sexual arousal group and two control groups. The second control group was recruited to examine possible confounding effects of the intertrial images in the second intentional binding task in two of the three groups (see *Design and procedure* section). Within the different groups, gender balance was given for the sexual arousal group (*N* = 34, *N*_*f*_ = 14, *N*_*m*_ = 17) and the neutral group with intertrial images (*N* = 31, *N*_*f*_ = 17, *N*_*m*_ = 14). The second control group without intertrial images differed in size and was comprised of more females than males (*N* = 25, *N*_*f*_ = 18, *N*_*m*_ = 7). There were no differences in age between the groups (sexual arousal *M* = 21.82 years, *SD* = 2.11, neutral with screenshots *M* = 22.00 years, *SD* = 2.44, neutral without screenshots *M* = 21.24 years, *SD* = 1.61, *F*(2, 86) = .955 *p* = .389).

Power analysis was performed post hoc using IBM SPSS Statistics 25 since the originally planned choice of analysis was re-evaluated and thus differed from the a priori chosen power analysis. Previous studies with a similar research question and design recruited 20 participants per condition (Christensen et al., 2019); we increased this number to 30 per condition. Testing for a two-way mixed ANCOVA (effect size *η*_*p*_^2^= .067, *α* = .05, three groups, two measures, sample size *N* = 90), a power = .692 was observed.

### Apparatus and stimuli

#### Intentional binding

The method for the Libet clock task (Haggard et al., 2002) was applied as a guiding procedure assessing intentional binding; the experimental paradigm was reproduced by the description of Aarts and van den Bos (2011). To program the experiment, a code with HTML5 Application Programming Interfaces (APIs) that maximizes accuracy and timing precision was modified. It included the following features: CSS animations for presenting visual stimuli, web audio API for presenting auditory stimuli, and DOM event timestamps for logging user interaction (Garaizar, Cubillas, & Matute, 2016).

**Fig. 1:**
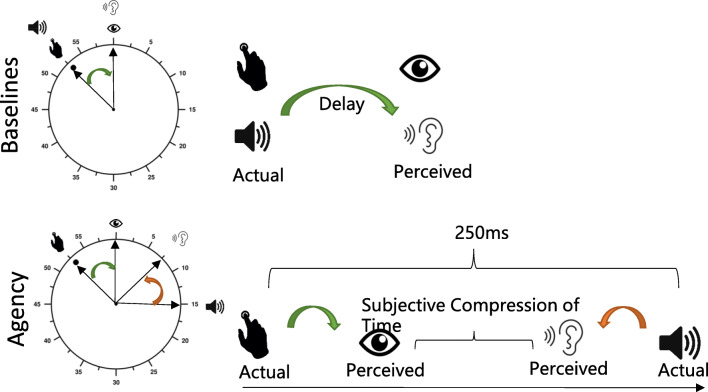
Intentional binding procedure

The experiment consisted of four different blocks: two baselines and two agency blocks. The participants watched an analogue clock with a rotation period of 2,560 ms, marked with numbers in intervals of five. In each trial, the clock rotated twice and the condition-specific event occurred in the second lap as the purpose of the first round was that they become used to the speed of the clock (Garaizar et al., 2016). In the *baseline action* condition, the participants watched the clock and pressed the space key during the second lap at a time of their choice. Afterwards, they were asked to report the position of the clock hand at the moment that they pressed the key. In the *baseline outcome* condition*,* the participants watched the clock and heard a tone at a random time. This time, they reported the position of the clock hand at the moment that they heard the tone. In the *agency action* and the *agency outcome* condition*,* the participants watched the clock and pressed the space key in the second lap at a time of their choice (as in the baseline action block), but this time a tone followed with a delay of 250 ms. Depending on the block (*agency action* and *agency outcome)*, the participants were asked to report the position of the clock hand at either the moment they had pressed the key or heard the tone (Fig. 1).

In the three conditions, when the participants had to press a key, they were asked not to press the key at a specific time (always at the same time or only at the interval marks of 5). In addition, participants were instructed to be as precise as possible (responses given in intervals of 1). All groups completed the experiment twice (pre- and post-emotion induction); each experiment contained four blocks with 20 trials (Moore et al., 2010). The presentation of blocks was randomized across participants.

### Questionnaires

#### Demographics

Participants reported gender, age, and sexual orientation.

#### Self-report of arousal and valence

**Fig. 2:**
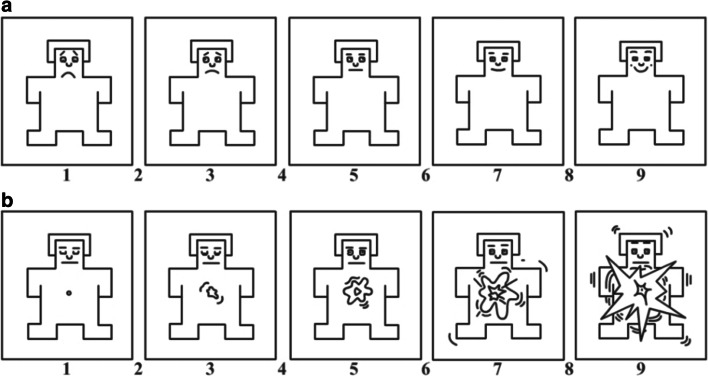
**a**, **b** Adapted version of the Self-Assessment Manikin (Lang, 1980) for valence and arousal ratings

Self-Assessment Manikin (SAM): A paper and pencil version of the SAM (Lang, 1980) was used to record self-experienced emotional and arousal states (Fig. 2a, b). Ratings were made on a 9-point Likert scale for valence and arousal (Carvalho, Leite, Galdo-Álvarez, & Gonçalves, 2012). Following Philippot (1993), participants were instructed to report what they had actually felt in response to viewing the film clip, rather than what they believed they should feel, and what they felt at the time that they viewed the film clip, not their overall mood. Since the SAM measures general arousal and not sexual arousal specifically, we added one item measuring the impact of sexual arousal in the sexual arousal condition (1 = not at all sexually arousing, 9 = very much sexually arousing).

### Procedure and experimental design

Sessions started with the SAM in paper-pencil form to assess self-reported valence and arousal, followed by the first computer-based intentional binding task. After completing the first part, participants watched a 6.5-min film clip showing either a sexually arousing scene (Threesome scene from the movie “Love,” 2015; Gaspar Noé) or a documentary film clip about the solar system/planets, depending on the group they were assigned to, and completed the SAM for arousal and valence afterwards for the second time. Subsequently, a post-induction of the intentional binding task was conducted. To ensure that sexual arousal was maintained after watching the film clip, intertrial images (screenshots) of the pornographic film clip were used in the second intentional binding block in the sexual arousal condition and intertrial images of the documentary clip were inserted in the second intentional binding task in one of the two control groups to guarantee comparability between the two groups. The second control group did not have intertrial images so that confounding effects of the images in general on binding could be controlled for. The total duration of the study was roughly 1.5 h.

### Statistical analysis

#### Control groups

Influence of Screenshots. Two two-way mixed-ANOVAs (between-factor “condition”; within-factor “time”) were used to investigate potential effects of the intertrial images from the two film clips.

#### Manipulation check

Manipulation check of emotional induction (dependent variable SAM scores) was done with a three-way mixed ANOVA between-factor “condition” (control without intertrial images, control with intertrial images, and sexual arousal group), and the within factors “emotion rating” (arousal, valence) and “time” (pre and post induction). T-tests for paired samples for arousal ratings adjusted with Bonferroni correction (*p* < .017) were used for post hoc analyses to examine the change over time in each condition.

#### Calculation of intentional binding

The intentional binding paradigm includes measures for binding of action (key, delay in the perception of action, and drift towards the time of tone) and binding of outcome (tone, earlier perception of tone, and drift towards the time of action) (Haggard et al., 2002). Perceived time was subtracted from actual time in each trial in order to determine the perception error, and medians for each of the four different blocks were used instead of means for each participant to eliminate outliers (Pockett & Miller, 2007). Action binding was calculated by subtracting the median in the baseline action block from the median in the agency action block (to generate the difference from baseline to the operant conditions). Therefore, smaller values represent greater action binding as it means the key was perceived to be closer to the tone. Outcome binding was calculated by subtracting the median in the agency outcome from the median in the baseline outcome minus block (Moore et al., 2010; Moore et al., 2011).

#### Main analysis

Analyses for influence of emotion induction on binding were conducted separately for each subcomponent due to the different pattern of action and outcome binding. Thus, two two-way mixed ANOVAs with the between-factor “condition” (sexual arousal, control with intertrial images, control without intertrial images) and within-factor “time” (pre- and post-induction) were conducted for action binding and for outcome binding individually. In addition, arousal ratings were integrated in the analyses to control for differences in arousal ratings between the groups. Therefore, the difference between post- and pre-arousal ratings were calculated for each participant and integrated as a covariate in two two-way ANCOVAs (between-factor “condition” and within-factor “time”), one for action binding and one for outcome binding, respectively. A Pearson correlation co-coefficient for arousal change (difference in post-arousal minus pre-arousal) and action binding change (difference in post-action binding minus pre-action binding) was used to interpret the interaction between action binding and arousal change.

## Results

### Control groups: Influence of intertrial images

Intertrial images of the neutral film clip did not influence the post-binding scores. Two two-way mixed ANOVAs (between-factor “condition”; neutral condition with vs. neutral condition without screenshot; within-factor “time”) were conducted, confirming control groups did not differ from one another, neither in action nor in outcome binding between the two intentional binding measurements (Table 1).

**Table 1:** Two two-way mixed ANOVAs with between-factor “condition” (neutral condition with vs. neutral condition without intertrial images) and within-factor “time”

	*F*(1, 54)	*P*	*η²*
Action binding			
Condition	.195	.660	.004
Time	.001	.973	.000
Interaction Time and Condition	1.525	.222	.027
Outcome binding			
Condition	.045	.883	.001
Time	.315	.577	.006
Interaction Time and Condition	1.031	.315	.019

### Manipulation check: Subjective ratings (SAM)

The means and standard deviations for SAM pre- and post-ratings of arousal and valence for each condition can be seen in Table 2. To assess sexual arousal induced by the erotic film clip specifically, participants in the sexual arousal group were also asked to what extent they evaluated the film clip as sexually arousing (*M* = 5.24 *SD* = 1.86); four participants chose not to answer this question.

**Table 2:** Means and standard deviations for pre- and post-arousal and valence ratings by condition (*N* = 90)

Condition		Pre	Post
Control without Intertrial images	Arousal	2.96 (1.86)	3.24 (1.90)
	Valence	6.44 (1.04)	6.00 (1.58)
Control with Intertrial images	Arousal	3.10 (1.90)	3.03 (1.97)
	Valence	5.84 (1.27)	5.94 (1.26)
Sexual arousal	Arousal	2.26 (1.19)	4.88 (1.92)
	Valence	5.94 (1.18)	5.71 (1.27)

The three-way mixed ANOVA (between-factor “condition”; within-factors “time” and “emotion rating” (arousal and valence)) showed significant main effects for time and emotion rating. Significant interactions were observed for time and condition, time and emotion rating and time, emotion rating and condition (Table 3).

**Table 3:** Three-way mixed with ANOVA between-factor “condition,” within-factors “time” and “emotion rating” (*N* = 90)

	*F*(2, 87)	*p*	*η²*_*p*_
Condition	.517	.598	.012
Time	5.632	.020	.061
Time and Condition	7.099	.001	.140
Emotion Rating	220.866	.000	.717
Emotion Rating and Condition	1.966	.146	.043
Time and Emotion Rating	26.810	.000	.236
Interaction Time, Emotion Rating, Condition	18.273	.000	.296

The three-way interaction shows a significant difference between pre- and post-rating in the sexual arousal condition (*t*(33) = -9.042 *p* < .001, pre *M* = 2.26 *SD* = 2.29, post *M* = 4.88 *SD* = 1.92), but no significant differences in the two control groups (control with intertrial images *t*(30) = .168 *p* = .868, pre *M* = 3.10 *SD* = 1.90, post *M* = 3.03 *SD* = 1.97, control without intertrial images *t*(24) = -.573 *p* = .572, pre *M* = 2.96 *SD* = 1.86, post *M* = 3.24 *SD* = 1.90). As sexual arousal is expected to be an ambivalent emotional state in a laboratory setting, an analogue increase of valence ratings was not predicted and could also not be demonstrated in our data.

### Main analysis

#### Action binding: Influence of condition

Results of the two-way mixed ANOVA (between-factor “condition”; within-factor “time”) did not confirm the hypothesis – no significant effects were found (Table 4). Action binding was not affected by sexual arousal specifically.

**Table 4:** Two-way mixed ANOVA with between-factor “condition” and within-factor “time” for action binding

	*F*(2, 87)	*p*	*η²*_*p*_
Condition	.119	.888	.003
Time	.520	473	006
Time and Condition	1.635	.201	.036

#### Action binding: Influence of arousal change

The differences between pre- to post-induction for the sexual arousal condition confirmed a successful manipulation of sexual arousal. Nevertheless, this did not guarantee comparability of arousal level in pre-inductions between the three groups. Hence, the change from pre- to post-arousal ratings was added to the analyses controlling for potential baseline differences independent of design. Post-arousal rating was subtracted from pre-arousal rating for each participant and included as a covariate. A two-way mixed ANCOVA with between-factor “condition,” within-factor “time,” and covariate “arousal change” revealed an interaction between time and arousal change on action binding (Table 5).

**Table 5:** Two-way mixed ANCOVA with between-factor “condition” and within-factor “time” and covariate “arousal change” for action binding

	*F*(2, 86)	*P*	*η²*_*p*_
Condition	.120	.887	.033
Time	.132	.717	.002
Time and Condition	.766	.468	.017
Time and Arousal Change	6.200	.015	.067

**Fig. 3:**
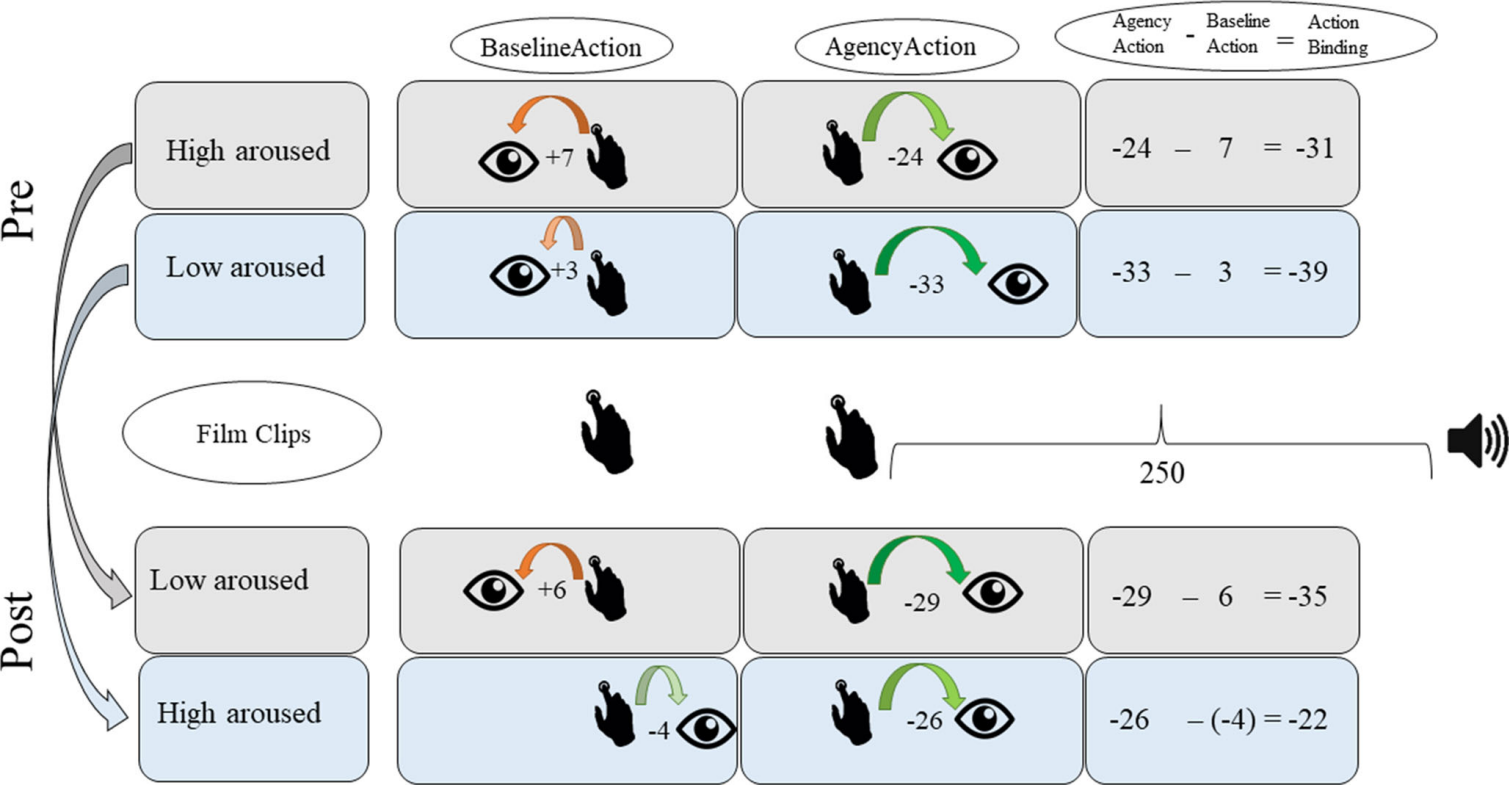
Action binding scores visualized by median split of arousal change, gray = decrease in arousal from pre to post induction, blue = increase in arousal from pre- to post-induction, numbers = mean perception error in ms, finger = actual key press, eye = perception of key press, speaker = actual tone

A Pearson correlation coefficient was used to clarify the direction of the effect between arousal change as a continuous measure and action binding with difference scores (pre-action binding was subtracted from post-action binding; pre-arousal rating was subtracted from post-arousal rating). These differences correlated positively with one another (*r* = .292 *p* = .005). Negative values in the arousal differences indicated a decrease in arousal from pre- to post-induction, whereas positive values indicated an increase in arousal over time. Smaller values represented greater action binding (key press was shifted towards tone). Hence, smaller values in the action binding difference indicated a stronger binding in the post-induction compared to pre-induction, whereas greater values indicate a weaker binding post-induction compared to pre-induction (key press was not shifted to tone). A positive correlation therefore implies that action binding is reduced in higher arousal and action binding is increased in lower arousal (Fig. 3).

#### Outcome binding: Influence of condition

The two-way mixed ANOVA (between-factor “condition”; within-factor “time”) for outcome binding did not show significant effects (Table 6). Outcome binding was still intact in the sexually aroused state.

**Table 6:** Two-way mixed ANOVA with between-factor “condition” and within-factor “time” for outcome binding

	*F*(2, 87)	*p*	*η²*_*p*_
Condition	1.615	.205	.036
Time	.174	.677	.002
Time and Condition	.508	.604	.012

#### Outcome binding: Influence of arousal change

Analogous to the analysis for action binding, arousal ratings were added as a covariate for outcome binding in order to control for potential differences in the first measurement of the intentional binding task between the three groups. A two-way mixed ANCOVA with between-factor “condition,” within-factor “time,” and covariate “arousal change” over time for outcome binding did not reveal significant effects (Table 7). Outcome binding seems to be independent of subjectively reported arousal level.

**Table 7:** Two-way mixed ANCOVA with between-factor “condition,” within-factor “time,” and covariate “arousal change” for outcome binding

	*F*(2, 86)	*P*	*η²*_*p*_
Condition	.123	.885	.003
Time	.050	.824	.001
Time and Condition	.548	.540	.013
Time and Arousal Change	.136	.713	.002

## Discussion

The goal of this study was to investigate the effect of sexual arousal on action binding in order to understand the underlying mechanisms of sexually deviant behavior. No significant effects have been found for sexual arousal specifically, but the current experiment supports the hypothesis that generally arousing states might be associated with a reduction in action binding measured with the Libet clock task. This pattern has previously been observed for emotionally negative arousing states such as fear and anger (Christensen et al., 2019). However, these findings are contrary to previous results for general arousal in the interval estimations task, which increased intentional binding (Wen et al., 2015). The inconsistency in results raised the question of whether the different kinds and/or extents of arousal impact intentional binding and its underlying processes in a different manner, or if this is caused by methodological differences between the studies. In line with the hypothesis, outcome binding was still intact in sexual arousal and not affected by general arousal either.

### Contribution to the research field

Although the effect of arousal on intentional binding has been examined before (Wen et al., 2015), our findings add to the current state of research as the inconsistency in results with previous results confirm methodological differences in the use of intentional binding tasks. Wen et al. (2015) used the interval-estimation task while this study was conducted with the Libet clock paradigm. It has previously been suggested that results of interval reproduction are determined by the perception of a causal relationship between two events, not taking into account if intentionality or agency are involved. This means, in the interval-estimation task, self-agency could modulate the perception of event boundaries while not modulating the perception of temporal intervals, which is not to be expected with the Libet clock procedure (Dewey & Knoblich, 2014). A meta-analysis comparing the two tasks revealed a larger effect size and higher sensitivity to perceptual moderators in binding observed with the Libet clock procedure than with the interval-estimation task (Tanaka et al., 2019).

Moreover, the increase in intentional binding of Wen et al. (2015) has not been linked directly to subjective ratings or objective arousal inductions of the same participants. Instead, ten different participants were recruited to evaluate their arousal levels induced by the red color of the jumping squares and to ascertain their skin conductance. That design assumes that the results of a small sample towards a subjective emotional response and objective skin conductance inductions can be generalized to a different sample. Although our design included subjective cognitive ratings only, arousal ratings of the participants were linked directly to their binding scores before and after the emotion induction. The differences in subjective emotion ratings between the participants within the same condition emphasize the importance of controlling for individual responses in emotional manipulation. For instance, individual differences such as personality and experience influence the response to emotional manipulation (Fisher & Byrne, 1978).

To conclude, our design offers an insight into the direct effects of an emotional state during action performance on action binding and outcome binding separately for the first time. While many studies have focused on manipulating the action outcome to investigate how much each subcomponent is affected by it, no study has differentiated between the effects of arousal on action and outcome binding individually. Our results add to the results of Christensen et al. (2019), who have reported a decrease of action binding in states of fear and anger, indicating that arousal has stronger effects than valence for the action binding processes in action performance.

### Dopamine hypothesis

Our results are in line with the view that action binding is more independent of temporal prediction than outcome binding, and that inaccurate predictions provide evidence of a specific impairment in action planning or generating action outcome predictions, rather than in the matching process of predicted and observed outcomes. As arousing states are associated with alterations in the dopaminergic pathways within different brain areas (Damsma, Pfaus, Wenkstern, Phillips, & Fibiger, 1992; Giuliano & Allard, 2001), a reduction in action binding during arousal reflects the changes in the dopaminergic system involved in action execution (Tanaka et al., 2019). Previous researchers have already highlighted dopamine as a determinant of intentional binding (Aarts et al., 2012; Graham, Martin-Iverson, & Waters, 2015; Moore et al., 2010). Several studies have found hints for an involvement of the dopaminergic system in intentional binding, such as ketamine as a model for psychosis (Moore et al., 2011), schizophrenia (Haggard, Martin, Taylor-Clarke, Jeannerod, & Franck, 2003; Hauser et al., 2011; Hur, Kwon, Lee, & Park, 2014; Voss et al., 2010), psychosis-like experiences, and age (Graham et al., 2015), as well as substance use (Render & Jansen, 2019). Patients with Parkinson’s disease, which is accompanied by a degeneration of dopamine-producing neurons causing disturbances in voluntary behavior, also showed a specific lack of action binding (Moore et al., 2010; Saito, Takahata, Murai, & Takahashi, 2015). It has been suggested that action binding results from cue integration (Wolpe, Haggard, Siebner, & Rowe, 2013). As uncertainty about the action effect increases, action binding is reduced, as cue integration cannot be executed. In Parkinson patients, experiencing unreliability in their motor execution (Caap-Ahlgren & Lannerheim, 2002), these uncertainties for action outcomes might cause a diminishment of action binding, aligned with reported deficits in sensorimotor integration. Outcome binding is driven by a different pre-activation mechanism, and not supposed to result from cue integration (Waszak, Cardoso-Leite, & Hughes, 2012; Wolpe et al., 2013). This may be the reason for the different results for action binding and outcome binding in Parkinson patients, and could also explain that outcome binding is still intact in arousal in this study.

### Limitations and future research

The results of the current study support the notion that general arousal, but not sexual arousal specifically, impairs action binding. If action binding is affected by general arousal, this could be relevant for models explaining violent behavior, as general arousal may be an important factor in sexual and general violent behavior. A next step could be to investigate if personality traits such as psychopathy that have a high prevalence in people committing offenses (Hare, Clark, Grann, & Thornton, 2000; Knight & Guay, 2006; Porter, Campbell, Woodworth, & Birt, 2001) show a different pattern of action binding in arousal. Including personality traits could elucidate if people with psychopathy have an impaired action binding in arousal or if they are less affected.

At this stage, our study design is restricted to behavioral results and self-reports and is not supported by more objective inductions on a physiological level. As we are in the phase of implementing a successful design, resource orientation was prioritized over recording any additional physiological data. Physiological data could be used to test the dopamine hypothesis or as a manipulation check of arousal. Spontaneous eye-blink rates, for example, can be used as a non-invasive indirect marker of central dopamine function (Jongkees & Colzato, 2016) and pupil dilation has been proposed as a reliable and valid marker of sexual arousal (Lick, Cortland, & Johnson, 2016). Physical arousal can also be measured in skin conductance as it is an independent indicator of sympathetic activity (Boucsein, 2012). Adding these measures would offer a reliable, valid method to examine arousal on different levels to link it to changes in intentional binding.

### Conclusion

Our study examines effects for arousing states whilst performing an action on both action and outcome binding separately for the first time. Previous research has focused on manipulating the consequences valence rather than the emotional state during the performance or reported results for intentional binding as a sum of action binding only. However, a recent meta-analysis (Tanaka et al., 2019) emphasizes the importance of reporting effects of both intentional binding subcomponents (action and outcome binding) in order to understand the underlying mechanisms in depths.

Our results are in line with previous research that showed a decrease in action binding in negative arousal such as fear and anger measured with the Libet clock task (Christensen et al., 2019), but are in contrast with previous results for general arousal in intentional binding measured with the interval-estimation task (Wen et al., 2015) suggesting a methods dependent variance. A decrease in action binding in aroused states could have implications for the comprehension of the underlying processes in violent behavior.

## Electronic supplementary material

ESM 1 (SAV 14 kb)
